# Aging and insulin signaling differentially control normal and tumorous germline stem cells

**DOI:** 10.1111/acel.12288

**Published:** 2014-12-03

**Authors:** Shih-Han Kao, Chen-Yuan Tseng, Chih-Ling Wan, Yu-Han Su, Chang-Che Hsieh, Haiwei Pi, Hwei-Jan Hsu

**Affiliations:** 1 Institute of Cellular and Organismic Biology, Academia SinicaTaipei, 11529, Taiwan; 2 Graduate Institute of Life Sciences, National Defense Medical CenterTaipei, 11490, Taiwan; 3 Department of Biomedical Science, College of Medicine, Chang Gung UniversityTao-Yuan, 333, Taiwan

**Keywords:** aging, cell cycle, GSCs, IGF, tumor, tumor stem cell

## Abstract

Aging influences stem cells, but the processes involved remain unclear. Insulin signaling, which controls cellular nutrient sensing and organismal aging, regulates the G2 phase of *Drosophila* female germ line stem cell (GSC) division cycle in response to diet; furthermore, this signaling pathway is attenuated with age. The role of insulin signaling in GSCs as organisms age, however, is also unclear. Here, we report that aging results in the accumulation of tumorous GSCs, accompanied by a decline in GSC number and proliferation rate. Intriguingly, GSC loss with age is hastened by either accelerating (through eliminating expression of Myt1, a cell cycle inhibitory regulator) or delaying (through mutation of *insulin receptor* (*dinR*) GSC division, implying that disrupted cell cycle progression and insulin signaling contribute to age-dependent GSC loss. As flies age, DNA damage accumulates in GSCs, and the S phase of the GSC cell cycle is prolonged. In addition, GSC tumors (which escape the normal stem cell regulatory microenvironment, known as the niche) still respond to aging in a similar manner to normal GSCs, suggesting that niche signals are not required for GSCs to sense or respond to aging. Finally, we show that GSCs from mated and unmated females behave similarly, indicating that female GSC–male communication does not affect GSCs with age. Our results indicate the differential effects of aging and diet mediated by insulin signaling on the stem cell division cycle, highlight the complexity of the regulation of stem cell aging, and describe a link between ovarian cancer and aging.

## Introduction

Stem cells undergo self-renewal to maintain tissues with rapid turnover, regenerate damaged tissues, and ensure optimal tissue and organ function. Stem cells are controlled by multiple layers of regulation, in response to local, systemic, and environmental factors (Drummond-Barbosa, 2008). Aging is a nearly universal process involving the deterioration of metabolic, muscular, reproductive, and cognitive functions, ultimately affecting lifespan. However, the molecular mechanisms by which aging alters stem cells are poorly understood.

**Figure 1:**
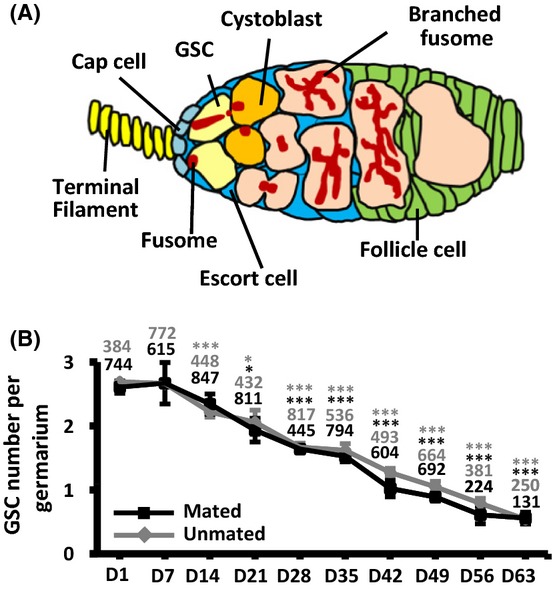
GSCs of mated and unmated females decline with age. (A) The *Drosophila* germarium houses 2–3 GSCs in the niche, which is formed of terminal filament cells, cap cells, and anterior escort cells. Each GSC contains a spectrosome (fusome). A single GSC division generates a cystoblast; this develops into a germ line cyst, which contains a branched fusome. The cyst is subsequently surrounded by somatic follicle cells. (B) Average GSC number per germarium decreases in mated and virgin females with age. D = days after eclosion. GSC numbers analyzed are shown above the error bar for each time point. *P < 0.05; ***P < 0.001. Error bars, mean ± SD

*Drosophila* is a small organism with a short lifespan; such properties, combined with the availability of powerful genetic approaches, facilitate the use of *Drosophila* in investigations of cellular responses at different physiological ages. Most importantly, the GSCs in the *Drosophila* ovary are well characterized (Fig. 1A; Kirilly *et al*., 2005), making it an excellent model in which to study how stem cells respond to aging. One *Drosophila* ovary is composed of 16–20 functional units, named ovarioles, which produce eggs (Spradling, 1993). The anterior-most structure of each ovariole (the anterior tip of the germarium) is the niche, which contains two to three GSCs. GSCs directly contact with niche cap cells, a major niche component, and the GSC fusome, an organelle with a membranous-like structure, is juxtaposed to the interface between cap cell and GSC. A single GSC division gives rise to a cystoblast, which undergoes four rounds of incomplete division to form a 16-cell cyst; the cells of the cyst are interconnected with branched fusomes (Spradling, 1993). The 16-cell cyst is then surrounded by a layer of follicle cells, and the entire structure buds off from the germarium and develops into a mature egg.

The insulin/insulin-like growth factor (IGF) signaling pathway is an evolutionarily conserved nutrient-sensing system (Barbieri *et al*., 2003), which is known to control stem cell division in many tissues(LaFever & Drummond-Barbosa, 2005; Speder *et al*., 2011). In *Drosophila*, the insulin pathway is activated by the binding of insulin-like peptides to their receptor, *dinr*. It has been previously shown that insulin signaling directly controls the G2 phase of the GSC division cycle in response to diet (Hsu *et al*., 2008). Notably, insulin signaling is attenuated in the ovary during aging (Hsu & Drummond-Barbosa, 2009), and the GSC division rate also decreases with age (Pan *et al*., 2007; Zhao *et al*., 2008). It is not clear, however, whether insulin signaling also controls the G2 phase of GSC cell cycle progression in response to aging.

Reproduction and longevity are closely linked, with signals between soma and germ lines coordinating the rate of organismal aging (Kenyon, 2010). In both *C. elegans* and *Drosophila*, elimination of germ cells extends lifespan by activating the insulin-signaling negative regulator, FOXO (Flatt *et al*., 2008). Conversely, other studies using *C. elegans* demonstrated that signals from the soma are coordinated with nutrient sensing to control reproductive status via insulin signaling (Luo *et al*., 2010). A more recent study reported that mating promotes GSC proliferation and oogenesis while reducing female lifespan in *C. elegans*, via communication between males and the female germ line and soma (Shi & Murphy, 2014). In *Drosophila*, mating promotes egg production by stimulating yolk protein accumulation in the oocytes, through effects mediated by male sex peptides (Soller *et al*., 1997). However, it is not clear whether mating in *Drosophila* affects GSC retention and division in response to aging.

In this study, we show that aging controls GSC division at S phase, in a process distinct from the dietary effect mediated by insulin signaling (which controls G2 phase progression of GSCs). However, insulin signaling is directly required for GSC maintenance with age. With age, DNA damage accumulates in GSCs, and induced tumorous GSCs escape the niche. Of note, GSC tumors which escape the niche continue to respond to aging in a similar manner to normal GSCs, indicating that physiological aging affects stem cell division via signals which act independently of the niche. In addition, we report that the decrease in the rate of GSC division with age may contribute to age-dependent GSC loss. Finally, we show that mating does not affect GSC maintenance or division in response to aging.

## Results

### GSC number is decreased with age, regardless of mating history

We initially investigated the effect of aging on GSCs. To this end, we carefully examined the number of GSCs in the niches of germaria at different ages (Fig. 1B), by identifying the anteriorly anchored fusomes (membranous cytoskeletal structures) of GSCs, which are juxtaposed to niche cap cells (Hsu *et al*., 2008). Newly eclosed flies carried 2.6 ± 0.1 GSCs (*n* = 744 germaria), and similar GSC numbers (2.7 ± 0.3 GSCs, *n* = 615 germaria) were observed in the niches of 7-day-old germaria. Gradual loss of GSCs was observed from the second week after eclosion. By 63 days after eclosion, the number of GSCs in germaria had dropped to 22% (0.6 ± 0.1 GSCs, *n* = 131 germaria) of that at 7 days after eclosion. Furthermore, half of the germaria in 56- and 63-day-old ovaries were degenerated. These results agree with previous reports that the number of GSCs significantly declines with age (Pan *et al*., 2007; Zhao *et al*., 2008) and is also consistent with the observation that *Drosophila* testicular GSCs undergo an age-related decline in number (Wallenfang *et al*., 2006). Mating may contribute to age-dependent GSC loss by exhausting GSCs, as mating increases the production of eggs (Pilpel *et al*., 2008), which are derived from GSCs. We observed similar GSC numbers in mated and unmated females at all time points, indicating that mating does not affect GSC maintenance during aging (Fig. 1B).

### The Fucci Cdt1 probe is not a valid G1 marker for GSCs, as it is present throughout the cell cycle

**Figure 2:**
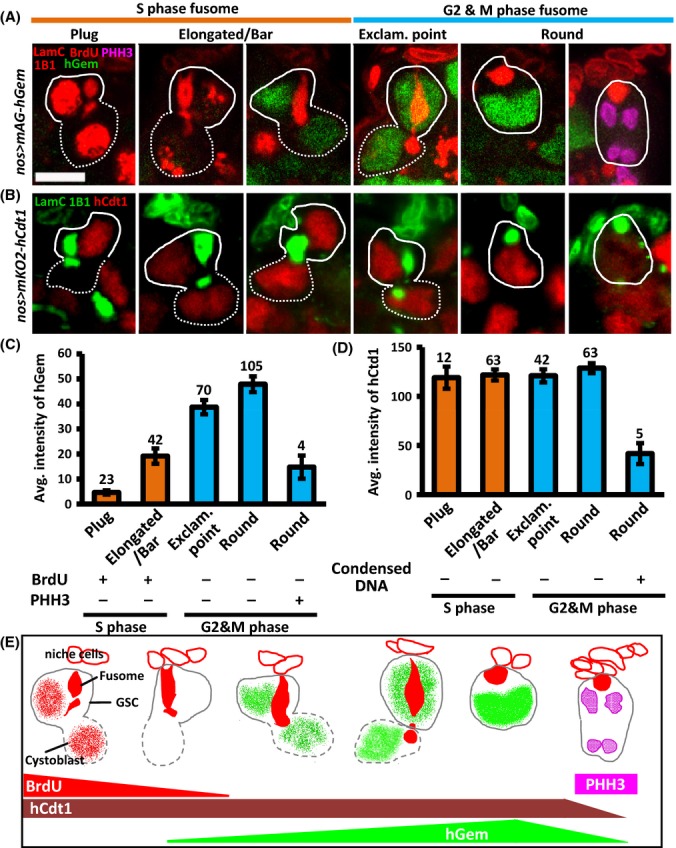
Detection of GSC cell cycle phase based on fusome morphology and cell cycle markers. (A and B) GSCs (solid lines) and their daughter cells (cystoblasts, dashed lines) in 7-day-old (A) germaria expressing human (h) Gem-mAG (green), and labeled with BrdU (red), PHH3 (magenta), 1B1 (red, fusomes), and LamC (red, terminal filament and cap cell nuclear envelopes), and (B) germaria expressing hCdt1–mKO2 (red), and labeled with 1B1 (green) and LamC (green). PHH3 and BrdU incorporation are markers for M and S phase, respectively. GSC fusomes are juxtaposed to cap cells, and these fusomes exhibit morphological changes during the cell cycle (Hsu & Drummond-Barbosa, 2009). During S phase, GSCs display a ‘plug’ (BrdU-positive) or ‘elongated/bar’ fusome morphology, as a nascent fusome (or plug) is assembled and then fused to the original fusome, thereby connecting the GSC and the cystoblast. During early G2, GSCs exhibit ‘exclamation (exclam.) point’ fusome morphology, as the connection between GSCs and the cystoblast is severed. During late G2 and M (PHH3-positive), GSC fusomes display a ‘round’ shape. Probe expression (hGem-mAG or hCdt–mKO2) is driven by *nos-GAL4*, a germ cell-specific driver. The scale bar is 5 μm. (C and D) Average (avg.) intensity of hGem (C) and hCdt1 (D) in GSCs at different cell cycle phases, as determined from fusome morphology, BrdU incorporation, PHH3, and condensed chromosomes (a characteristic of M phase) in (A and B). GSC numbers analyzed are shown above each bar. Error bars, mean ± SD (E) A scheme for the correlation between GSC fusome morphology and expression of cell cycle markers during S/G2/M in GSCs.

Several lines of evidence indicate that the ability of stem cells to proliferate decreases with age (Jones & Rando, 2011). To determine how GSC division is affected by aging, it was first necessary to identify appropriate cell cycle markers. GSC fusome morphology changes periodically during GSC division and has been used for analyzing GSC cell cycle progression (de Cuevas & Spradling, 1998; Hsu *et al*., 2008; LaFever *et al*., 2010; Ables & Drummond-Barbosa, 2013). During G1/S phase, the GSC fusome elongates to fuse with the newly synthesized fusome of its daughter cell (cystoblast), resulting in a ‘plug’ and subsequently ‘elongated/bar’ fusome morphology (see Fig. 2A). Of note, the GSC and cystoblast are synchronized throughout S phase, and therefore, BrdU was distributed over the same portion of the nucleus in every labeled pair of cells. Cytokinesis between the GSC and its daughter cell is complete by early G2 phase, and the GSC fusome is pinched off to produce an ‘exclamation point’ morphology; the fusome then becomes ‘round’ during G2/M (see Fig. 2A). However, the lack of validated G1 markers (Hsu *et al*., 2008; Ables & Drummond-Barbosa, 2013) prevents discrimination between S and G1 phase GSCs.

To identify a suitable marker, we introduced transgenic fluorescent ubiquitination-based cell cycle indicator (Fucci) probes into the germ line (described in the Materials and Methods). Fucci probes are composed of the ubiquitination domain of human Cdt1 and Geminin (Gem) fused to mKO2 (monomeric Kusabira-Orange 2) and mAG (monomeric Azami-Green), respectively (Sakaue-Sawano *et al*., 2008). The mKO2–hCdt1 probe accumulates during G1 phase (producing orange cells), while mAG-hGem accumulates during S/G2/M phase (producing green cells); this has previously been observed in somatic cells, including those of *Drosophila* (Nakajima *et al*., 2011). We characterized GSC cell cycle progression based on GSC fusome morphology, PHH3 (an M phase marker), BrdU incorporation (an S phase marker), and Fucci probes (Fig. 2). We observed that the expression profile of hGem during GSC cell cycle progression was similar to that of somatic cells (Fig2A,C, and E). hGem was extremely weak in GSCs carrying ‘plug’ fusomes (hereafter, we consider such low expression to be equivalent to no expression), but it exhibited higher expression in GSCs carrying ‘elongated/bar’ fusomes. Expression of hGem peaked at G2 phase, but then declined when GSCs entered mitosis (PHH3-positive GSCs with rounded fusomes). However, unlike somatic cells, hCdt1 was highly expressed throughout the GSC cell cycle, except for during M phase (characterized by the presence of condensed chromosomes; Fig. 2B,D, and E). This expression pattern differed to that of *Drosophila* Cdt1 (double parked; dup), which exhibits negligible expression in GSCs displaying G1/S phase fusome morphology and strongest expression at G2 phase (Ables & Drummond-Barbosa, 2013). Nevertheless, neither *Drosophila* Cdt1 nor the Fucci probe, hCdt1, can serve as a G1 marker for GSCs.

### GSCs exhibit an extremely short G1 phase

We thus identified GSCs at G1 phase by the absence of PHH3 (M phase), BrdU (S phase), and hGem (S/G2/M phase) signals (Table 1). We scored GSCs (*n* = 520) in 7-day-old germaria by examining the expression of those cell cycle markers and fusome morphology. However, we did not detect such GSCs through our scoring, implying that G1 phase is extremely short during GSC division, in agreement with previous reports (Hsu *et al*., 2008; Ables & Drummond-Barbosa, 2013). Surprisingly, all GSCs with ‘plug’ fusomes exhibited high incorporation of BrdU and no hGem expression, while only 14.7% of GSCs (*n* = 150) with ‘elongated/bar’ fusomes contained punctate BrdU signals (see also Fig. 2A); this finding indicates that ‘plug’ and ‘elongated/bar’ GSC fusome morphology represent early and late S phase, respectively. Based on this finding and the expression profile of hGem in GSCs (see Fig. 2), we conclude that the GSC fusome morphology previously defined as G1/S phase primarily represents S phase.

**Table 1:** GSCs exhibit an extremely short G1 phase

Cell cycle phase	Expression of cell cycle markers	Fusome morphology	% of GSCs	
M	PHH3^+^/BrdU^−^/Gem*	Round		2.3
G1	PHH3^−^/BrdU^−^/Gem^−^	‡		0
S	PHH3^−^/BrdU†/Gem^−^	Plug	4.4	
	PHH3^−^/BrdU*/Gem*	Elongated/Bar	4.2	33.3
	PHH3^−^/BrdU^−^/Gem*	Elongated/Bar	24.6	
G2	PHH3^−^/BrdU^−^/Gem†	Exclamation point	17.1	
	PHH3^−^/BrdU^−^/Gem†	Round	47.3	64.4
Total GSCs			520	

GSCs with S phase fusome morphology	GSC number	% of GSCs positive for BrdU labeling
Plug	23	100 (23)§
Elongated/Bar	150	14.7 (28)

Flies expressing human Geminin (Gem) under the control of a germ cell-specific driver, *nos-GAL4*, were cultured at 25°C until dissection. Food was changed daily. The germaria of one-week-old flies expressing Gem were labeled with PHH3 (M phase marker), BrdU (S phase marker), 1 B1 (GSC fusomes), and LamC (GSC niche cells). The germaria were then scored for GSCs with different combinations of cell cycle markers and fusome morphology

* Represents weak expression

† Represents strong expression.

‡ Fusome morphology was not identified.

§ The numbers of GSCs positive for BrdU labeling are shown in parentheses.

### Aging delays S phase progression in GSCs

**Figure 3:**
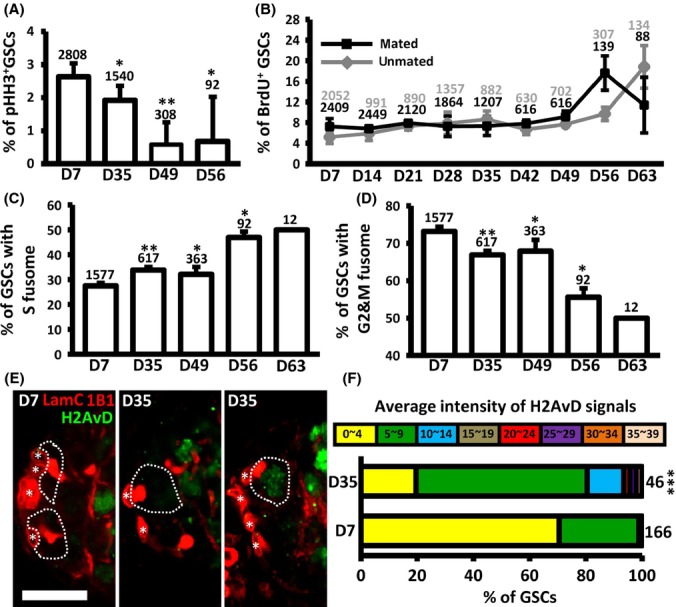
GSCs exhibit delayed S phase progression and accumulation of DNA damage during aging. (A) Detection of PHH3 reveals that the frequency of GSCs in M phase decreases with age. (B) Detection of BrdU-positive (+) cells reveals that the percentage of GSCs in S phase does not exhibit changes from day (D)7 to D63. (C) The percentage of GSCs with ‘S’ phase fusome morphology increases with age. (D) The percentage of GSCs with ‘G2 & M’ phase fusome morphology decreases with age. GSC numbers analyzed are shown above each bar. *P < 0.05; **P < 0.01, ***P < 0.001. Error bars, SD (E) Anterior structures of 7-D-old and 35-D-old germaria labeled with H2Av (a DNA damage marker, green), 1B1 (red, fusomes), and LamC (red, cap cell and terminal filament cell nuclear envelopes). Scale bar, 10 μm. Dashed circles mark GSCs. (F) The percentage of GSCs at each average H2AvD signal intensity at D7 and D35. GSC numbers analyzed are shown beside each bar.

We proceeded to compare the cell cycle profiles of GSCs of *yw* control flies at different ages through examining GSC fusome morphology and measuring the frequency of M (PHH3) and S phase (BrdU) cells (Fig. 3). The frequency of PHH3-positive GSCs in 56-day-old females (0.7 ± 1.2, *n* = 92) was reduced 4-fold as compared to those in 7-day-old females (2.6 ± 0.4, *n* = 2808, *P *< 0.05) (Fig. 3A); this result indicates that GSC division rate decreases with age in female flies, in agreement with previous reports (Pan *et al*., 2007; Zhao *et al*., 2008). We further examined whether aged GSCs exhibit less division activity in *vivo* by BrdU retention assay (Fig. S1A); enhanced division would result in increased consumption of BrdU, and vice versa. To this end, we fed 7- and 35-day-old flies on a BrdU-containing diet for 24 h and then examined BrdU levels in flies dissected immediately (considered as day 0), and 2 and 4 days after BrdU feeding. At 4 days after BrdU feeding, only 25% of the original amounts of BrdU (82.2 ± 4.3 arbitrary units) were retained in young GSCs (*n* = 149), while aged GSCs (*n* = 134) still contained 75% of the original amounts (27.4 ± 10.3 arbitrary units) (Fig. S1B–G); this indicates that the cell cycle of aged GSCs is approximately 3 times longer than that of young GSCs.

A decline of GSCs in M phase should also be reflected by fewer GSCs undergoing DNA replication. However, age did not affect the frequencies of BrdU-positive GSCs (which mainly represent GSCs at early S phase), and this was also unaffected by mating (Fig. 3B). Similar results were observed in male GSCs; the proportion of GSCs at M phase decreased with age (Wallenfang *et al*., 2006), while the frequency of GSCs at S phase was unaffected (Fig. S2). Our results suggest that aging delays the S phase progression of GSCs, accounting for the lack of a decrease in the proportion of BrdU-positive GSCs.

We then measured the relative length of time that GSCs of different ages spend in the S and G2 phases (Fig. 3C and D). In young females (7-day-old), approximately 27.5 ± 1.2 and 72.5 ± 1.2% of GSCs (*n* = 1577) exhibited ‘S’ and ‘G2/M’ phase fusome morphology, respectively, reflecting the relatively long G2 phase of GSC division (Table 1) (Hsu *et al*., 2008; LaFever *et al*., 2010; Ables & Drummond-Barbosa, 2013). Compared to young GSCs, the percentage of GSCs (*n* = 92) in 56-day-old germaria with ‘S’ fusome morphology was increased to approximately 45 ± 7.6% (*P *< 0.05), whereas the percentage of GSCs displaying ‘G2/M’ fusome morphology was decreased to 58.1 ± 7.0% (*P *< 0.05). These data indicate that aging predominately slows down S phase progression in GSCs, through a process distinct from the regulation of G2 phase by insulin signaling (Hsu *et al*., 2008).

### DNA damage accumulates in GSCs with age

DNA damage is known to be induced by aging (Chen *et al*., 2007), and slow S phase progression (Kumar & Huberman, 2004), suggesting that DNA damage may accumulate in GSCs with age. To address this hypothesis, we analyzed DNA damage levels in GSCs of young and aged females by examining the pattern of γH2AvD (Madigan *et al*., 2002), a marker of DNA damage analogous to mammalian γH2AX (Fig. 3E). Expression of γH2AvD was increased in 35-day-old GSCs as compared to 7-day-old GSCs (*n* = 166) (Fig. 3F). Furthermore, we detected γH2AvD in mitotic cysts (yellow arrow heads) of both young and aged germaria (Fig. 3E), suggesting that γH2AvD foci are also generated in response to replication stress (Mehrotra *et al*., 2008). DNA damage could also lead to cell death; however, we did not detect any apoptotic GSCs at different ages (Fig. S3), suggesting that aged GSCs with accumulated DNA damage may just leave the niche and undergo differentiation, although we cannot rule out the possibility that GSCs may die in different ways. Our results suggest that aging induces DNA damage in GSCs, and this is accompanied by prolonged S phase progression.

### Aging induces tumor-like GSCs and enlarged and mislocated niche cells in the ovary

**Figure 4:**
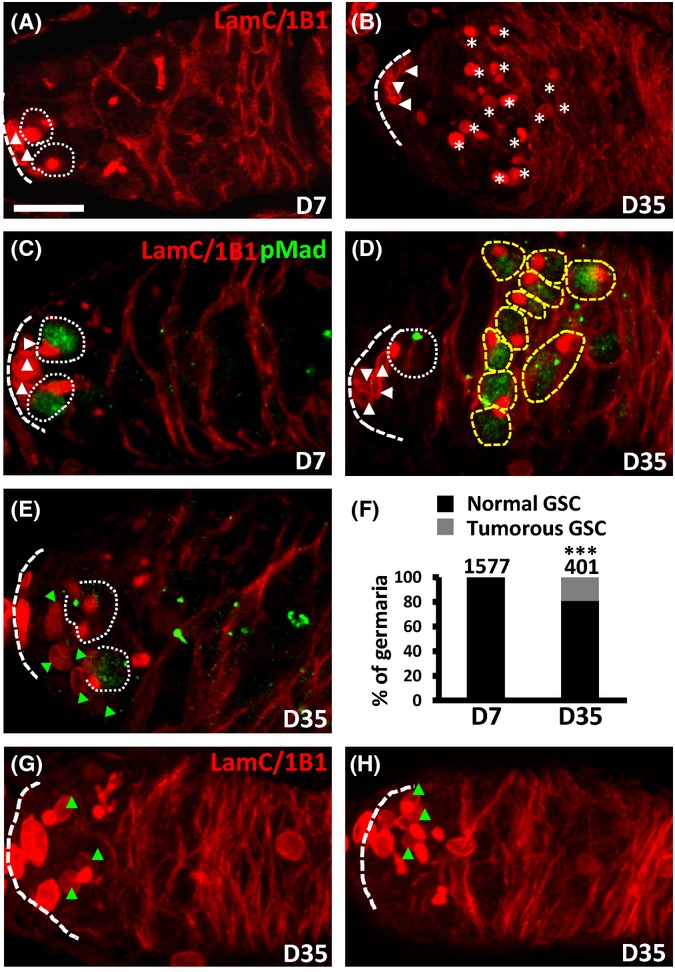
Tumorous GSCs and enlarged and mispositioned niche cells are induced in aged germaria. (A-E) 7-day (D)-old (A and C) and 35-D-old (B, D, E) germaria labeled with 1B1 (red, fusomes) and LamC (red, terminal filament, and cap cell nuclear envelopes); panels (C-E) also show labeling against the stemness factor pMad (green). Dashed lines mark the anterior edge of the germarium. White dashed circles mark normal GSCs in normal niche cap cells (white triangles); yellow dashed circles mark tumorous GSCs; and asterisks mark tumorous GSC spectrosomes. Scale bar, 10 μm. (F) Percentage of germaria carrying normal and tumorous GSCs at D7 and D35. GSC numbers analyzed are shown above each bar. ***P < 0.001. (G and H) 3D reconstructions of images from 35-D-old germaria labeled with 1B1 (red, fusomes) and LamC (red, terminal filament, and cap cell nuclear envelopes). Green triangles indicate enlarged and mislocated niche cells.

DNA damage often causes genome instability, which is highly associated with tumorigenesis (Mills *et al*., 2003). We observed tumor-like GSCs in aged germaria (Fig. 4). In young germaria (7-day-old, *n* = 1577) (Fig. 4A,C, and F), two or three GSCs were observed in the germarial anterior tip, followed by differentiating cyst cells bearing branched fusomes. However, 20% of 35-day-old germaria (*n* = 401) contained germ cells with ‘round’ fusome morphology, which occupied the posterior of germaria (asterisks) (Fig. 4B,D and F), suggesting these germ cells possessed undifferentiated, tumor-like properties. Both the age-induced tumor-like germ cells and young GSCs expressed comparable levels of phospho (p)-Mad (Fig. 4C and D), a mediator of self-renewal Dpp signaling and a marker for GSCs in wild-type (Kai & Spradling, 2003), indicating that the undifferentiated germ cells are tumorous GSCs. As expected, 35-day-old GSCs exhibited decreased p-Mad expression (Fig. 4D and E). We also observed fewer tumor-like GSCs in 56-day-old germaria (data not shown), which may be attributed to age-dependent degeneration of germaria. In addition to the accumulation of tumorous GSCs, 36.8% of 35-day-old germaria (*n* = 87) carried large and mispositioned niche cells (green triangles), as evidenced by the presence of LamC in nuclear envelopes and their attachment with GSCs (Fig. 4G and H). These aged niche cells were distal from the germarial anterior tip, which is the usual location of niche cells in young germaria (white triangles, *n* = 87 germaria). We frequently observed aged germaria carrying mislocated niche cells without tumorous GSCs, or vice versa, indicating the independency of these two phenomena. Remarkably, in addition to the *yw* strain, tumorous GSCs were also induced in aged ovaries of other wild-type strains, including *w*^*1118*^, Canton-S, and Oregon R (Fig. S4). Our results indicate that the formation of tumorous GSCs and large and mispositioned niche cells is an age-dependent process.

### Insulin signaling and GSC cell cycle progression contribute to GSC maintenance with age

**Figure 5:**
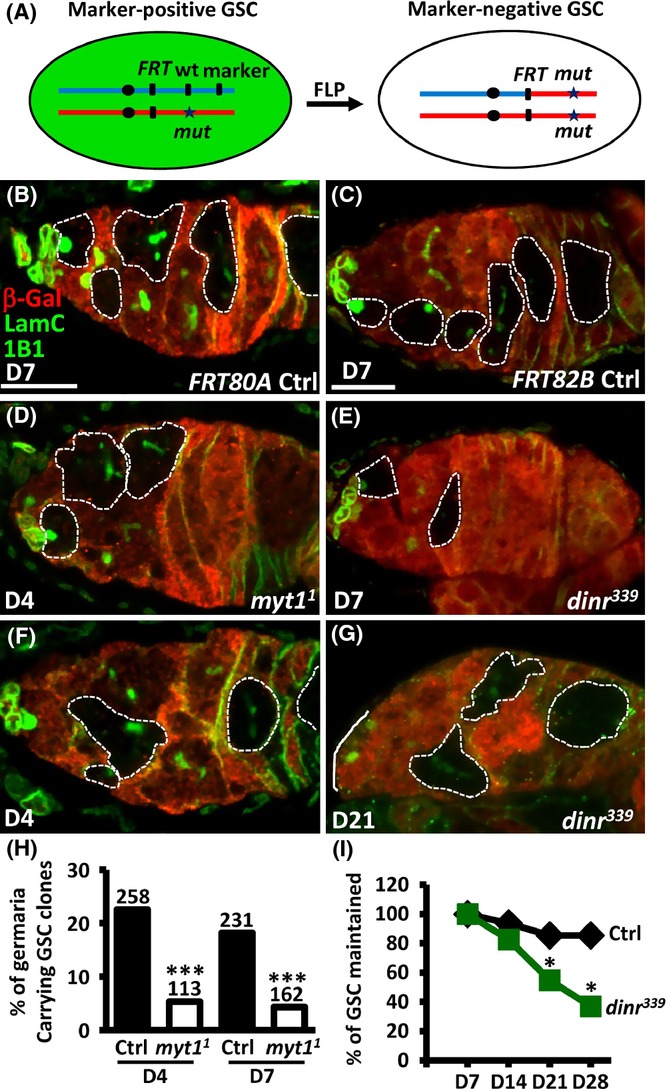
Altering the rate of GSC division disturbs GSC maintenance. (A) The FLP/FRT system. In females carrying a wild-type allele (wt) linked to a marker gene *in trans* with a mutant allele (*), FLP-mediated recombination between the FRT sites during mitotic division generates a homozygous mutant cell recognized by the absence of marker expression. (B-G) Control (Ctrl) (B and C) and *myt1*^*1*^ mutant (D and F) and *dinr* ^*339*^ mutant mosaic germaria (E and G) labeled with β-gal (red, wild-type cells), 1B1 (green, fusomes), and LamC (green, cap cell nuclear envelopes). GSCs and their progeny are outlined by dashed circles. Scale bars, 10 μm. The solid line in G marks the edge of the germarium. (H) Percentages of germaria carrying GSCs homozygous for *myt1*^*1*^ at 4 and 7 days (D4 and D7) after clone induction. Numbers of germaria are shown above each bar. ***P < 0.001. (I) Relative percentages of germaria carrying GSCs homozygous for *dinr*^*339*^ at the indicated days after clone induction.

We subsequently investigated whether decreased GSC division contributes to age-induced GSC loss. We specifically altered GSC proliferation using Flipase (FLP)/Flipase recognition site (FRT)-mediated mitotic recombination (Xu & Rubin, 1993) and examined how this affected GSC retention (Fig. 5 and Table S1). *Drosophila* Myt1 is a cdk1 inhibitor kinase which regulates multiple steps of cell cycle progression; *myt1* mutant GSCs exhibit faster cell cycle progression (Jin *et al*., 2005). Insulin signaling directly controls GSC division, and *dinr* mutant GSCs exhibit delayed cell cycle progression (de Cuevas & Spradling, 1998; Tseng *et al*., 2014). We therefore generated single mutation *myt1*^*1*^ and *dinr*^*339*^ GSCs (recognized by the absence of β-gal signals) (Fig. 5A–D and F–H) and examined their maintenance with age. We first confirmed that *myt1*^*1*^ mutant GSCs divided faster than normal GSCs by counting the number of control versus mutant progeny (cystoblasts and cysts) present in *myt1*^*1*^ mutant mosaic germaria containing at least one mutant GSC [because each progeny is derived from one GSC division, the ratio of mutant to wild-type progeny is a measure of their relative division (LaFever & Drummond-Barbosa, 2005)]. The number of progeny derived from control GSCs expressing β-gal was approximately equal to those without β-gal expression in *FRT* control mosaic germaria, indicating a relative division rate equal to approximately 1.0 at 4 and 7 days after clone induction (ACI) (Fig. S5 and Table S1). In agreement with a previous report (Jin *et al*., 2005), *myt1*^*1*^ mutant GSCs divided faster than their neighboring control GSCs (∼1.7-fold) (Fig. S5 and Table S1). To determine whether *myt1*^*1*^ mutant GSCs with faster division would be lost faster than those that divide slower in the germarium, we analyzed the proportion of germaria carrying at least one *myt1*^*1*^ mutant GSC at 4 and 7 days ACI. At 4 days ACI, 22.5% of control germaria (*n* = 258) carried β-gal-negative control GSCs, while only 5.3% of germaria (*n* = 113) (Fig. 5E) carried *myt1*^*1*^ mutant GSCs. Between 4 and 7 days ACI, *myt1*^*1*^ mutant GSCs decreased slightly (Fig. 5E and Table S1). We suspected that GSCs may be rapidly lost from their niche ACI. Indeed, 25.8% of *myt1*^*1*^ mosaic mutant germaria contained no *myt1*^*1*^ mutant GSCs, but did carry *myt1*^*1*^ mutant germ cell cysts (recognized by the absence of β-gal signals) at 4 days ACI (Fig. 5D); therefore, at least one *myt1*^*1*^ mutant GSC had been lost from these germaria. In contrast, only 10% of control mosaic germaria lost GSCs (*P *< 0.01). Interestingly, *dinr*^*339*^ mutant GSCs also decreased with age (Fig. 5I). At 4 weeks ACI, 88.2% of control germaria (*n* = 235) still carried cloned GSCs, while only 35.1% of *dinr*^*339*^ mosaic germaria (*n* = 224) carried GSCs (*P *< 0.05). These results indicate that Myt1 and insulin signaling, which are involved in the regulation of GSC cell cycle progression, also affect GSC maintenance. These data also imply that GSC division may contribute to age-dependent GSC loss, a hypothesis supported by earlier findings that mutation of CycA, CycB, or Cdc25 in GSCs results in GSC loss (Wang & Lin, 2005; Chen *et al*., 2009; Inaba *et al*., 2011).

### Interactions with a normal niche are not required for the age-induced decline of GSC proliferation

It is not known whether delayed GSC division in response to aging requires normal niche architecture. The self-renewal Dpp signals produced by the niche are required for GSC maintenance via suppression of the differentiation factor Bam (Kirilly *et al*., 2005). Removal of *bam* function results in the accumulation of GSCs outside of the niche. We found that GSCs within the tumors of *bam* mutant ovaries still respond to aging; as for normal GSCs, aging also decreased the proportion of *bam* GSCs entering M phase without affecting the proportion of GSCs positive for BrdU labeling (Fig. S6). These results indicate that tumor GSCs may respond to aging in a similar manner to normal GSCs.

### FACS-based DNA histograms from GSCs exhibit an inverted pattern to those of other cell types

**Figure 6:**
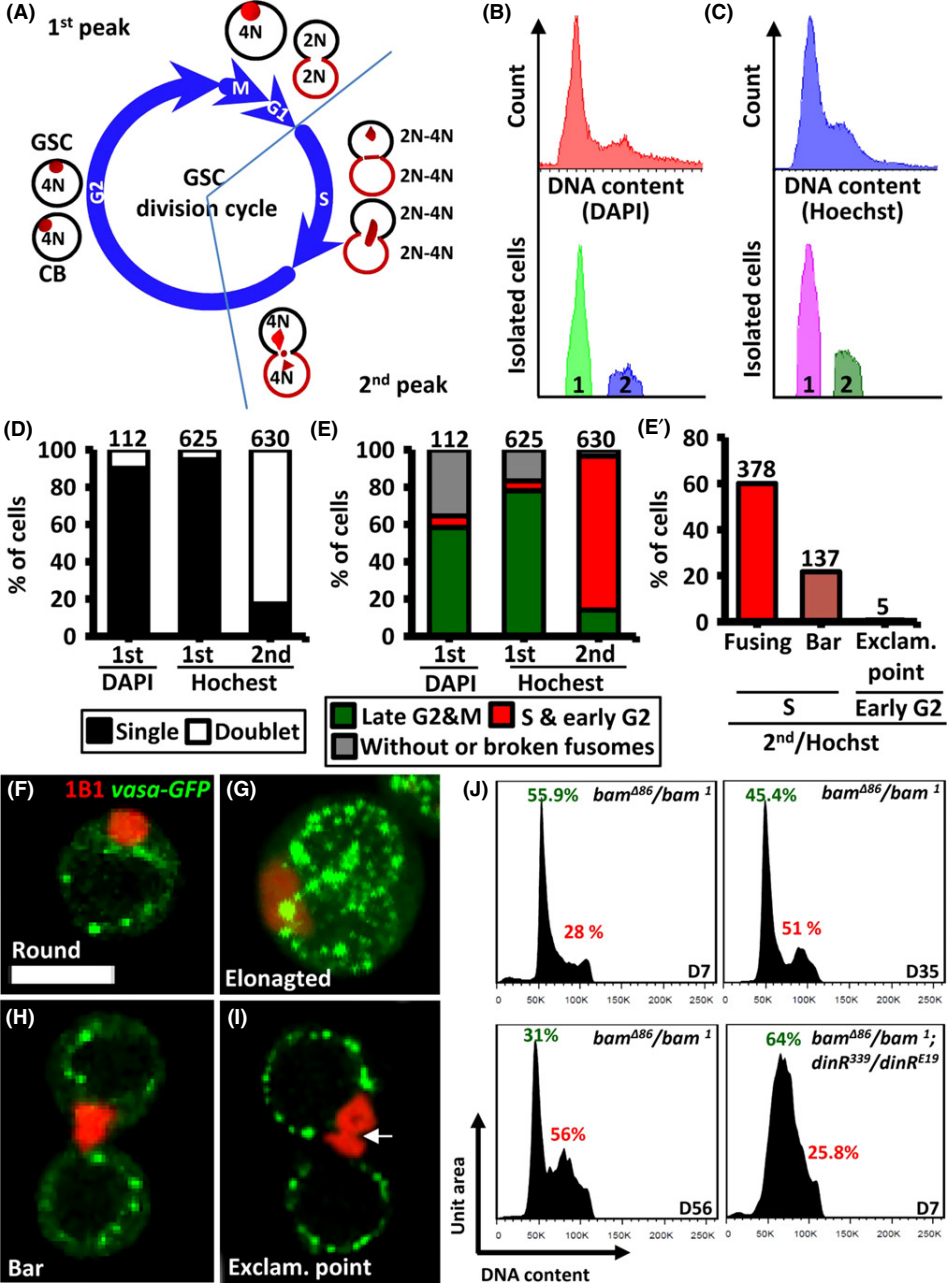
Tumorous GSCs exhibit unique DNA content histograms, and a delay in S phase progression in response to aging. (A) A scheme of the GSC division cycle. During M phase, GSCs possess ‘round’ fusomes and carry 4 copies of each chromosome (4N). After M phase, the GSC remains connected with its daughter cell (cystoblast; CB), forming a doublet cell until late G2 phase. At G1 phase, fusome morphology is not defined; the double cell carries two copies of each chromosome (2N). During S phase of the next division cycle, the doublet cell carries an elongating fusome with four to eight copies of DNA (4 to 8N), due to DNA replication. During early G2, the doublet cell exhibits an ‘exclamation point’ fusome, as the connection between the GSC and the cystoblast is severed. Later in G2, the GSC fusome becomes ‘round’ again. During G2, both the GSC and its daughter cell carry four copies of each chromosome. (B and C) FACS-based DNA content analyses of tumorous GSCs isolated from 7-day (D)-old *bam*^*1*^*/bam*^*Δ86*^ transheterozygous mutant ovaries by DAPI (B) and Hoechst (C) staining. Peaks ‘1′ and ‘2’ represent the cells sorted from DAPI- (B) or Hoechst-(C)labeled GSCs. (D) Numbers of singular (black bars) or doublet GSCs (white bars) isolated from the 1st peak of DAPI-labeled GSCs or from the 1st and 2nd peak of Hoechst-labeled GSCs. (E) Numbers of GSCs carrying late G2/M phase fusomes (rounded fusomes, green bars) or early G2/S phase fusomes (elongated fusomes, red bars) in the 1st peak of DAPI-labeled GSCs or the 1st or 2nd peaks of Hoechst-labeled GSCs. (E’) Numbers of GSCs carrying S phase (elongated and bar) or early G2 phase (exclamation point) fusome morphology in the 2nd peak of Hoechst-labeled GSCs. GSC numbers analyzed are shown above each bar. (F to I) Sorted Hoechst-labeled GSCs carrying *vasa-GFP* transgenes (green, germ cells) and labeled with 1B1 (red, fusomes), exhibiting round (F), elongated (G), bar (H), or exclamation (exclam.) point (I) fusomes. G is a 3D reconstruction. The arrow indicates the pinching off of the fusome by the closed ring canal upon completion of cytokinesis. Scale bar, 10 μm. (J) Cell cycle analyses of GSCs isolated from *bam*^*1*^*/bam*^*Δ86*^ transheterozygous mutant ovaries of the indicated ages, or in a *dinr*^*E19*^*/dinr*^*339*^ transheterozygous mutant background at D7.

To further confirm the above findings, we introduced a *vasa–GFP* transgene into the tumor GSCs of *bam* mutant germaria and analyzed cell cycle progression using FACS to measure cell DNA content (Fig. 6). DNA histograms usually contain two peaks; the first peak represents the number of cells with 2 copies of each chromosome at G1/G0 phase, while the second peak represents the number of cells with four copies of each chromosome at G2/M phase. However, in the GSC DNA histogram, the first peak represented cells at G1/late G2/M phase, and the second peak represented cells at S phase and early G2 phase (Fig. 6A–E). This occurs due to GSCs remaining in contact with their daughter cells (cystoblasts) after M phase, thereby forming a doublet cell until early G2 phase (Fig. 6A). As a result, the copy number of each chromosome in a doublet cell is 4, 4–8, and 8 at G1, S, and early G2 phase, respectively. After early G2 phase, the GSC separates from its daughter cell, thereby becoming a singular cell containing 4 copies of each chromosome again until the end of M phase. We evidenced this hypothesis by sorting cells from the DAPI- or Hoechst-labeled GSCs of the first and second peaks of the DNA histogram (Fig. 6B and C, respectively), by determining whether they were singular or doublet cells by immunostaining (Fig. 6D), and by establishing their cell cycle phase based on their fusome morphology (Fig. 6E–E'). We found that DAPI staining was better than Hoechst staining at separating the two DNA peaks. On the other hand, GSCs stained with Hoechst exhibited fewer disruptions in morphology after sorting; this may be because fixed cells (for DAPI staining) are more easily damaged during sorting. Most of the GSCs isolated from the first peaks of GSCs labeled with DAPI and Hoechst were singular and carried G2/M phase fusomes (Fig. 6D–F). Due to the low abundance of GSCs in the second peak and damage to cell morphology, we were unable to isolate GSCs from the second peak of DAPI-labeled cells. However, 81% and 0.7% of GSCs from the second peak of Hoechst-labeled GSCs exhibited late S and early G2 phase fusome morphology, respectively (Fig. 6E, G–I). Notably, exclamation point fusome morphology was apparently shorter in tumor GSCs than in normal GSCs (Fig. 6I). Our results indicate that the first peak of the GSC DNA histogram represents GSCs in G2/M phase, while the second peak is primarily comprised of GSCs in S phase.

### Tumor GSCs respond to aging in a similar manner to normal GSCs

Finally, we examined how tumor GSCs respond to aging, by analyzing their DNA content with DAPI staining at different ages and in the *dinr* mutant transheterozygous background (*dinr*^*E19*^*/dinr*^*339*^), respectively (Fig. 6J). From 7 to 56 days after eclosion, the percentage of tumor GSCs at G2/M phase decreased from 55.9% to 31%, suggesting an increase in the rate of G2 phase progression; meanwhile, tumor GSCs at S phase increased from 28% to 56%, indicating that S phase progression was delayed. In contrast, the percentage of mutant GSCs at G2 phase was increased, while the percentage at S phase was decreased, in a 7-day-old *dinr*^*E19*^*/dinr*^*339*^ background, a finding similar to that previously reported (Hsu *et al*., 2008). Our results reveal that S phase progression of tumor GSCs, like that of normal GSCs, is delayed by aging and is independent of regulation by insulin signaling.

## Discussion

In this study, we dissected how *Drosophila* female GSCs respond to age. Specifically, we have shown that aged GSCs exhibit a delay at S phase of the GSC division cycle, while insulin signaling-defective GSCs exhibit a prolonged G2 phase; this suggests that age-responsive signals affect the division of stem cells independently of insulin signaling. We previously demonstrated a role for insulin signaling in supporting GSCs with age via the maintenance of niche integrity (Hsu & Drummond-Barbosa, 2009); here, we demonstrate that insulin signaling directly controls GSC maintenance, indicating that this pathway acts both intrinsically and extrinsically to control stem cells. We also demonstrate that age is associated with GSC loss, DNA damage accumulation, and tumor formation. Further, aging-mediated disruption of GSC cell cycle progression may affect GSC maintenance, although the mechanisms involved are not clear. Moreover, an intact niche or male–female germ line communication is not required for GSC maintenance or division with age. Finally, we also show that tumor GSCs respond to aging in a similar manner to normal GSCs.

### DNA damage may be the cause of S phase delay in GSCs during aging

Although aging results in a decline in stem cell proliferation, relatively few studies have addressed how stem cell cycle progression is altered by aging. DNA damage is mainly induced by by-products of cellular metabolism, such as reactive oxygen species (ROS) and environmentally induced lesions upon irradiation. Accumulation of irreversible genomic DNA damage has been implicated as a prominent cause of aging, both at the organismal and at the cellular levels (Jung & Brack, 2014). Cells respond to DNA damage by activating checkpoint pathways, which delay cell cycle progression and allow for repair of the defects. Here, we observed that aged GSCs exhibit accumulation of DNA damage and a prolonged S phase, suggesting that the former may be responsible for the latter in GSCs during aging. DNA breaks result in activation of ATM/ATR kinases (ataxia-telangiectasia mutated and Rad3 related), which phosphorylate a variant of histone H2A (H2AX); this histone variant is a critical factor in facilitating the assembly of specific DNA-repair complexes on damaged DNA (Zou, 2007). ATM/ATR kinase-mediated signaling is part of the intra-S phase checkpoint pathway, and its activation is often associated with a delay in S phase progression (Bartek *et al*., 2004). However, *ATR* heterozygous mutant (*mei-41*^*D3*^*/+*) GSCs still exhibited a similar degree of S phase delay compared to wild-type, suggesting that ATR may be dispensable for age-induced S phase delay, although it is possible that disruption of one copy of *ATR* may not be sufficient to block the intra-S check point pathway (Fig. S8A). To our surprise, we observed a 65% increase of aged *tufe*^*atm−8*^*/+* GSCs in S phase (1.98-fold increase relative to young *tufe*^*atm−8*^*/+* GSCs), as compared to its sibling controls at the same age (1.33-fold increase relative to young control GSCs) (Fig. S8B). Coincidently, a recent publication on *Drosophila* reported that ATM functions in DNA damage repair and exerts negative feedback control over the level of programmed double strand breaks (DSBs) during meiosis, and thus the number of H2AX foci (a marker of DNA damage) is dramatically increased in *tufe*^*atm−8*^ mutant germ cells (Joyce *et al*., 2011). We speculate that *tufe*^*atm−8*^*/+* GSCs may induce more DNA damage via feedback regulation, thereby causing more severe S phase delay. However, in mice, *Atm*^*−/−*^ undifferentiated spermatogonia are not maintained in the testis due to DNA damage-induced cell cycle G1 arrest (Takubo *et al*., 2008), suggesting that ATM may function in the G1 phase in response to DNA damage. Nevertheless, it remains to be elucidated whether ATM mediates different cell cycle regulators in different cell contexts or in response to different types of stress-induced DNA damage.

### Dedifferentiation may be involved in the process of aging-mediated induction of tumorous GSCs

With age, cells may accumulate DNA mutations that allow them to escape normal regulatory processes and become tumor cells. Although tumorigenesis is harmful to health in the long term, it may also serve as a survival and protective mechanism when the body is highly threatened. While the germarium normally houses differentiating 8- or 16-germ cell cysts interconnected with branched fusomes, here, we found that the middle portion of the aged germarium was occupied by tumor-like GSCs, which express pMad (a Dpp signaling effector) and possess rounded fusomes. This result recalls an earlier report that forced stemness Dpp signaling causes differentiating germ cell cysts to revert into functional stem cells in *Drosophila* ovaries, through the induction of ring canal closure and fusome scission (Kai & Spradling, 2004). It has also been reported that aged human epidermal cells can dedifferentiate into stem cell-like cells via Wnt/β-catenin signaling (Zhang *et al*., 2012), and injury can drive the dedifferentiation of epidermal cells via the β-integrin-mediated signaling pathway (Li *et al*., 2013); these findings suggest that dedifferentiation is a process by which organisms address aging or tissue damage. Given that GSCs play a fundamental role in producing the next generation, we suspect that these tumor-like GSCs may be derived from germ cell cysts through a dedifferentiation process triggered by aging; however, we cannot rule out the possibility that these tumor-like GSCs are derived from the transformation of normal GSCs.

### Mating is dispensable for GSC division and maintenance in *Drosophila*

In invertebrates, including *C. elegans* and *Drosophila*, mating is detrimental to the lifespan of females, to increase progeny production (Paukku & Kotiaho, 2005; Flatt *et al*., 2008). In *Drosophila*, mating females die earlier than unmated females, and sex peptides, produced from the male accessory gland, may be responsible for this effect (Wigby & Chapman, 2005). In *C. elegans*, females shrink and die after mating, and this is partially due to the stimulation of GSC proliferation by sperm (Shi & Murphy, 2014). In this study, however, we did not observe differences in GSC proliferation rates between mated and unmated females at any age, suggesting that the promotion of GSC proliferation by mating may be specific to *C. elegans*. In addition, our results also indicate that sex peptides do not affect GSCs, at least at the level of proliferation. Moreover, we also observed similar rates of aging-induced GSC loss in mated and unmated females, suggesting that mating does not affect the physiological status of GSCs.

## Experimental procedures

Experimental procedures can be found in Data S1.

## Supporting Information

**Fig. S1** Age decreases GSC division rate. **Fig. S2** The proportion of Male GSCs at the S phase examined by EdU incorporation is not decreased with age. **Fig. S3** Age induces apoptosis in germarial germ cell cysts and somatic cells. **Fig. S4** Age induces accumulation of tumorous GSCs in the ovary of different strains. **Fig. S5***myt1*^1^ mutant GSCs divide faster than normal GSCs. **Fig. S6** Tumor GSCs respond to aging similarly to normal GSCs. **Fig. S7** Isolation of tumor GSCs from adult ovaries. **Fig. S8** Disruption of one copy of *atm*, but not *atr*, further delays the S phase progression of the GSC division cycle. **Table S1***myt1* and *dinr* are required for GSC maintenance. **Data S1** Experimental Procedures.
