# Supplementary material for: Aging and insulin signaling differentially control normal and tumorous germline stem cells
Source: Aging Cell. 2014 Dec 3;14(1):25–34. doi: 10.1111/acel.12288 (PMC4326914; doi:10.1111/acel.12288)
Supplement: Supplementary file 1 [file acel0014-0025-sd1.pdf]

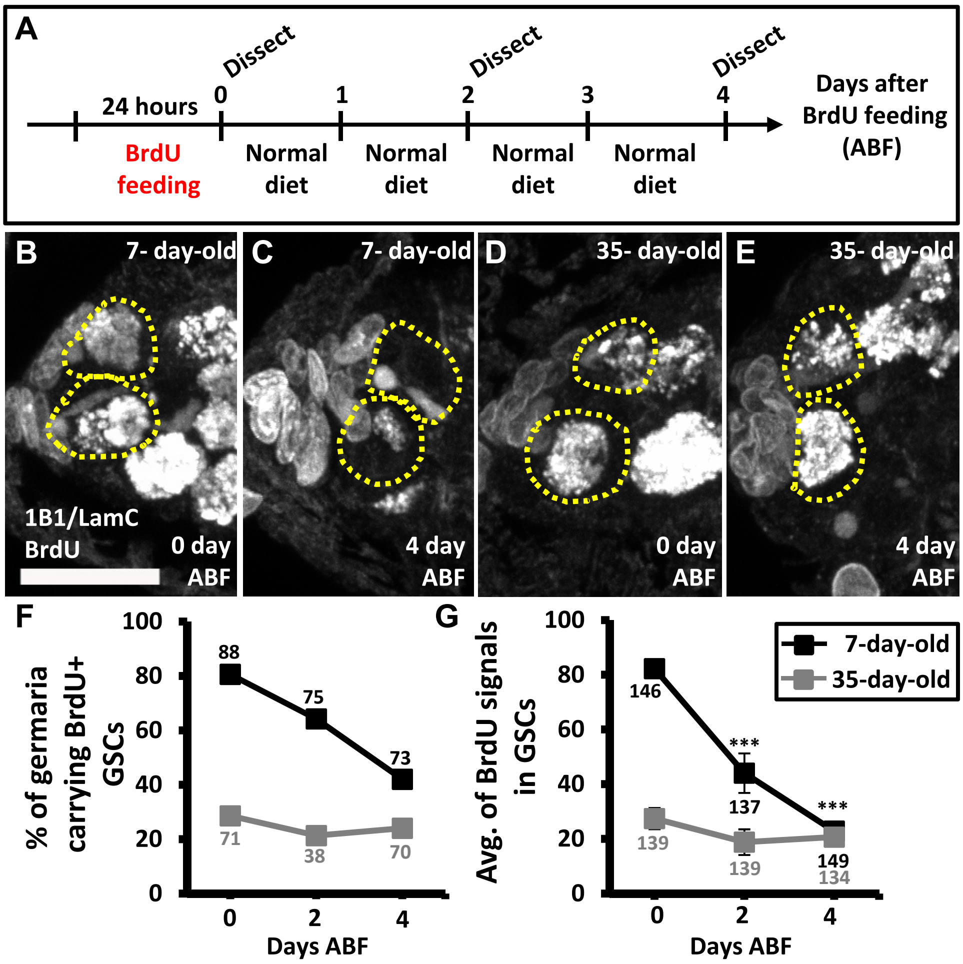

Supplementary Fig. 1 Age decreases GSC division rate . (**A**) A scheme depicting the BrdU retention assay. Seven- and 35-day-old flies were fed on BrdU-containing food for 24 hours, and then switched to normal food. Flies were dissected immediately after BrdU feeding (ABF), 2 days ABF, or 4 days ABF. (**B-E**) 3D reconstruction images of 0 (**B** and **D**) and 4 days ABF germaria (**C** and **E**) from 7- (**B** and **C**) and 35-day-old flies (**D** and **E**) labeled with 1B1 (gray, fusomes), LamC (gray, cap cell nuclear envelops), and BrdU (gray, DNA). Yellow dashed lines outline GSCs. Scale bar, 10 μm. (**F**) Percentage of germaria carrying BrdU-positive (+) GSCs, and (**G**) Average (Avg.) of BrdU intensity in GSCs at different time points ABF. Number of germaria (**F**) and GSCs (**G**) analyzed are shown below each square symbol.

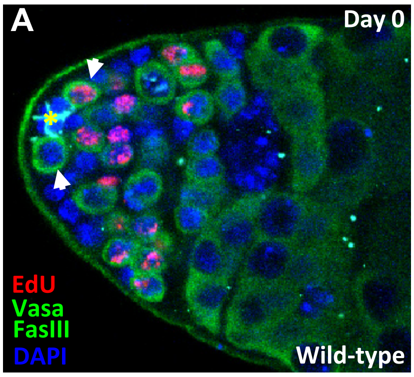

B. Proportion of GSCs positive for EdU incorporation

Total number	Day 0	Day 10	Day 30
testes	16	22	10
GSCs	136	167	66
EdU^+GSCs	29	27	14
EdU index (%)¶	21.3	16.2	21.2

¶ Percentage of EdU^+GSCs in total GSCs analyzed.

Supplementary Fig. 2 The proportion of Male GSCs at the S phase examined by EdU incorporation is not decreased with age. **(A)** Newly eclosed wild-type w^{1118} male testis labeled with Vasa (green, germ cells), FasIII (green, niche hub cells), and EdU (red). Arrows point GSCs that directly contact with niche hub cells; Asterisk indicates niche hub cells. (**B**) EdU index in male GSCs at Day 0, Day 10 and Day 30. Chi-Square analysis reveals no significance decrease of EdU index with age.

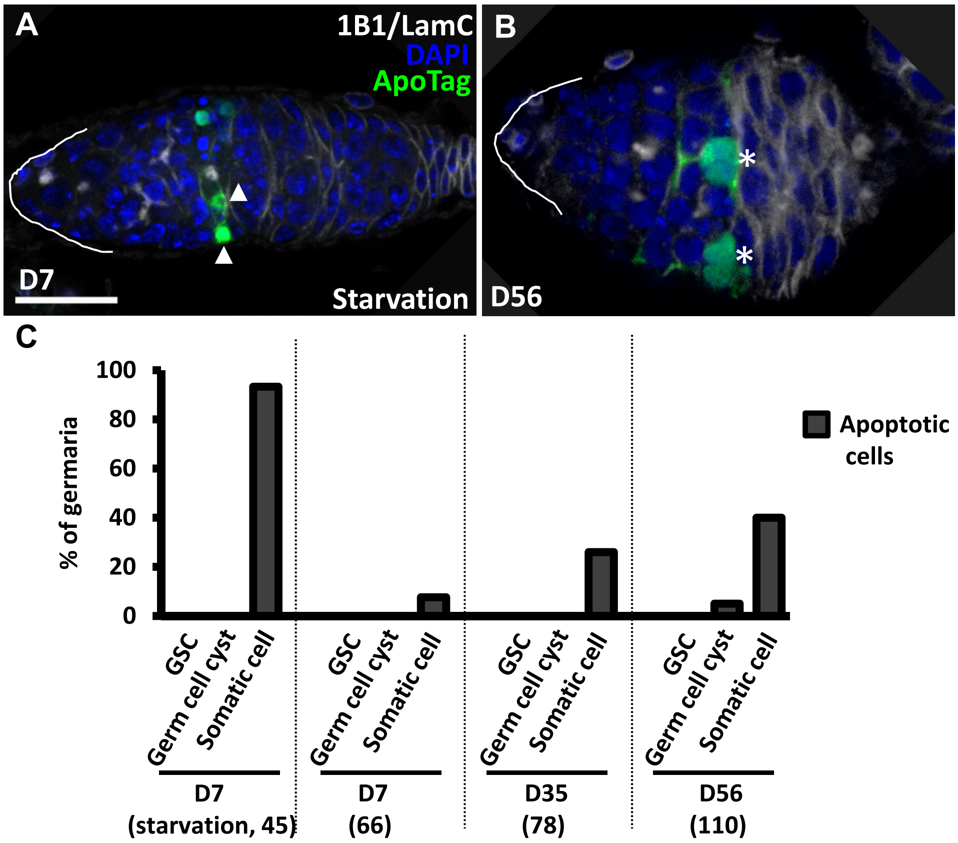

Supplementary Fig. 3 Age induces apoptosis in germarial germ cell cysts and somatic cells. (**A** and **B**) Starved 7-day(D)-old (**A**) and normal 56-D-old (**B**) germaria labeled with 1B1 (gray, fusomes), LamC (gray, cap cell nuclear envelopes), Apotag (green, apoptotic cells), and DAPI (blue, DNA). Solid lines mark the anterior edge of germaria. Arrow heads point apoptotic somatic cells and asterisks point apoptotic germ cell cysts. Scale bar, 10 μm. (**C**) Percentage of germaria carrying apoptotic GSCs, germ cell cysts, or somatic cells at indicated ages under starvation or normal conditions. Numbers of germaria analyzed are shown in parentheses.

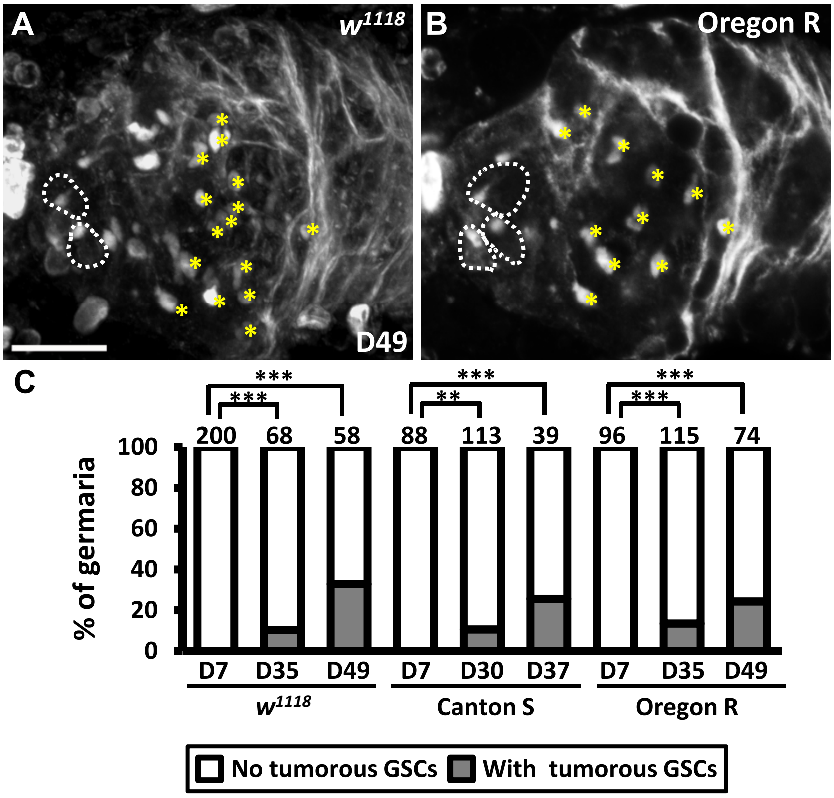

Supplementary Fig. 4 Age induces accumulation of tumorous GSCs in the ovary of different strains. (**A**) 3D reconstruction images of 49-day(D)-old *w^{1118}* (**A**) and Oregon R (**B**) germaria labeled with 1B1 (gray, fusomes), and LamC (gray, cap cell nuclear envelopes). Round fusomes (indicated by asterisks) within germaria are a sign of undifferentiating germ cells. Scale bars, 10 μm. GSCs are outlined by dashed lines. Note that no Canton S survived to D49.(**B**) Percentage of germaria carrying tumorous GSCs. The number of germaria analyzed is shown above each bar. **, P<0.01; ***, P<0.001. Statistical analyses were performed using the Chi-square test.

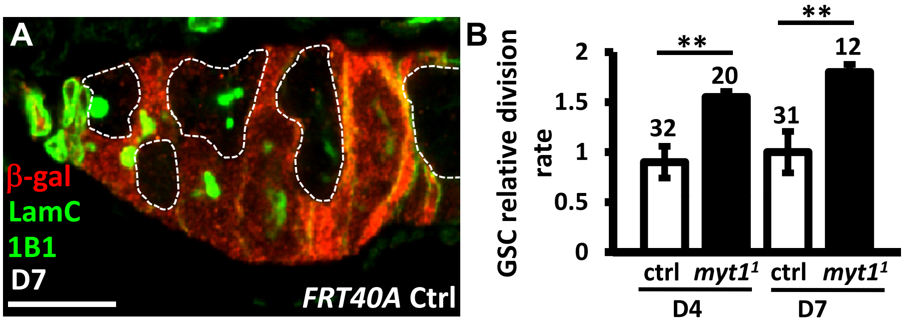

Supplementary Fig. 5 *myt1*^{1} mutant GSCs divide faster than normal GSCs. (**A**) 7-day(D)-old control (Ctrl) mosaic germaria labeled with β-gal (red, wild-type cells), 1B1 (green, fusomes), and LamC (green, cap cell nuclear envelopes). GSCs and their progeny are outlined by dashed circles. Scale bar, 10 μm. The same image shown in Fig. 5B. (**B**) Relative division of *myt1*^{1} mutant GSCs at 4 days and 7 days after clone induction. The number of GSCs analyzed is shown above each bar. Error bars, mean ± SEM. **, *P*<0.01.

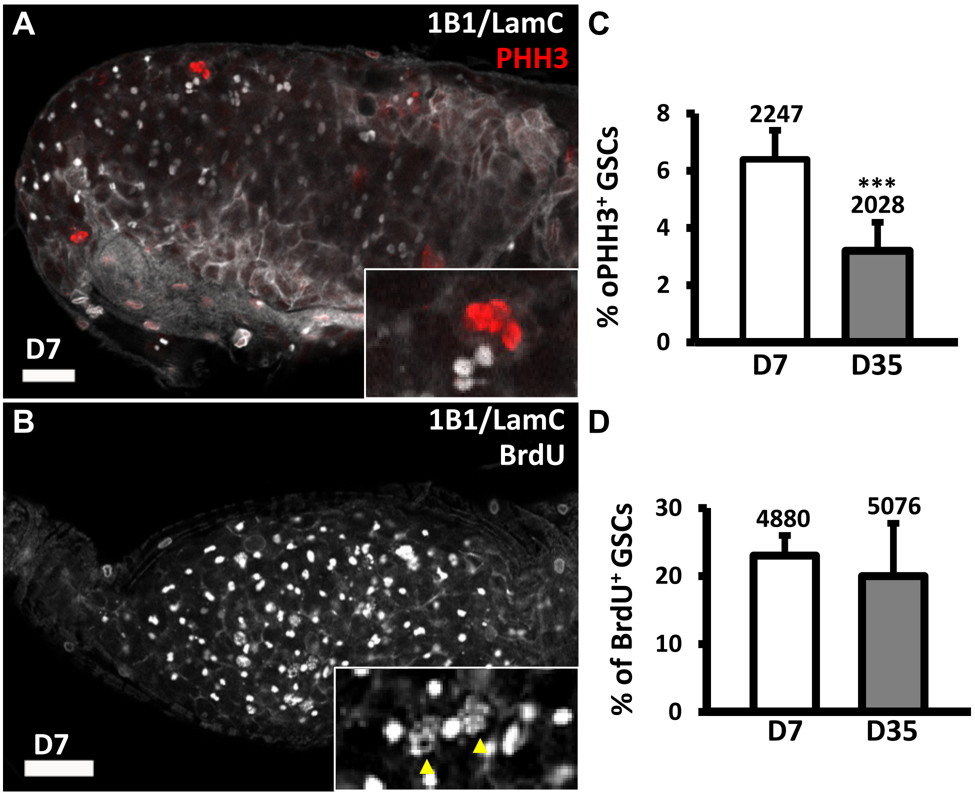

Supplementary Fig. 6 Tumor GSCs respond to aging similarly to normal GSCs. (A and B) *bam*$^{\Delta 86}$/*bam*^{1} germaria labeled with PHH3 (red) (**A**), BrdU (gray) (**B**), and 1B1 (gray, fusomes). Examples of PHH3-positive and BrdU-positive (arrow heads) GSCs are shown in inserts under a higher magnification. (**C**) the frequency of GSCs in M phase within *bam*$^{\Delta 86}$/*bam*^{1} tumors decreases with age, as shown by the decreased percentage of PHH3-positive GSCs. **(D)** Detection of BrdU-positive (+) cells reveals that the percentage of *bam*$^{\Delta 86}$/*bam*^{1} tumor GSCs in S phase does not exhibit changes from day(D)7 to D35. Scale bars, 20 μm. Number of GSCs analyzed are shown above each bar; bars in graphs show standard deviation. Asterisk, $P < 0.001$.

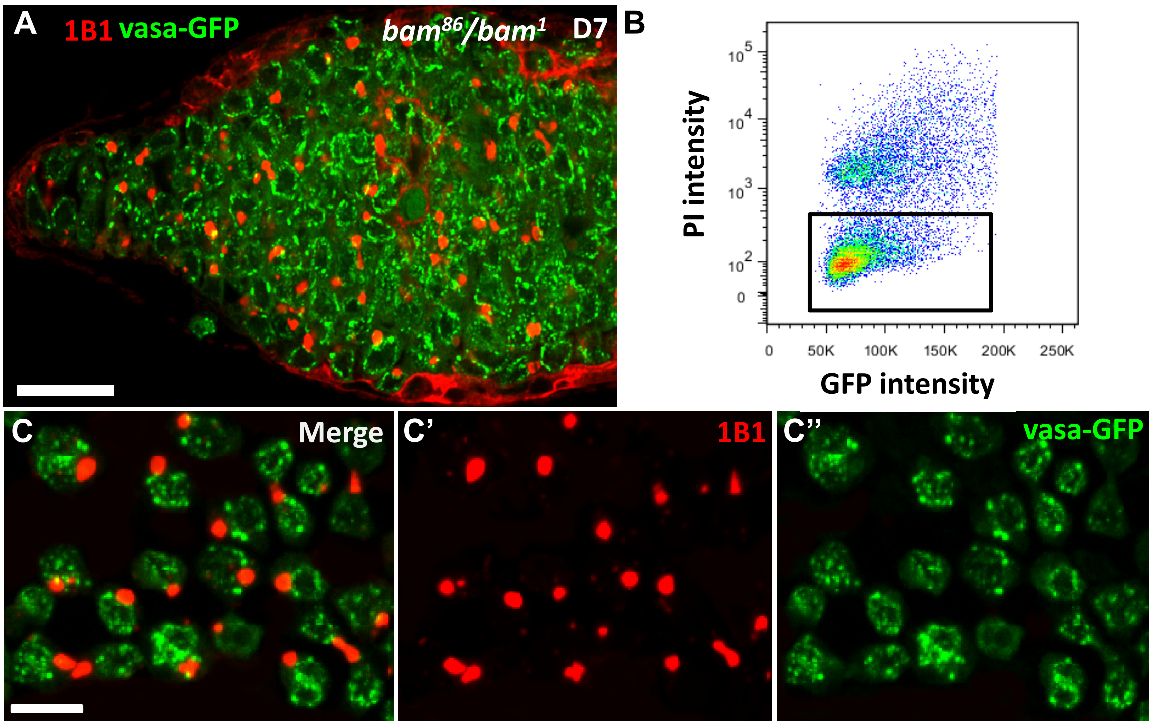

Supplementary Fig. 7 Isolation of tumor GSCs from adult ovaries. (A) *bam$^{\Delta 86}$/bam^{1}* germaria expressing the germ cell marker vasa-GFP (green) and labeled with 1B1 (red, fusomes). **(B)** FACS profile of cells from *bam$^{\Delta 86}$/bam^{1}*, *vasa-GFP* ovaries. GSCs were isolated based on high expression of GFP and negative for PI staining, which marks dead cells **(C-C")** Purified GSCs labeled with 1B1 (red). Scale bars, 20 μm **(A)** and 10 μm **(C)**.

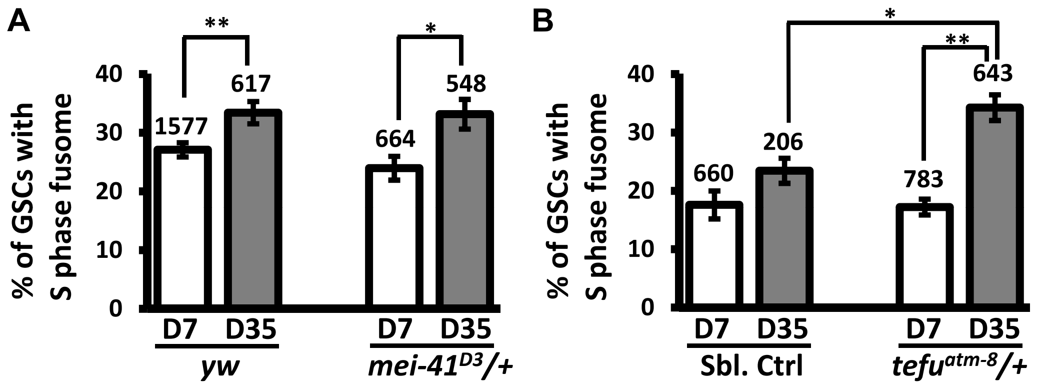

Supplementary Fig. 8 Disruption of one copy of *atm*, but not *atr*, further delays the S phase progression of the GSC division cycle. **(A and B)** Percentage of GSCs with S phase fusome in (**A**) *yw*, *atr* heterozygous mutant (*mei-41*^{D3}/+), (**B**) *atm* heterozygous mutant (*tefu*$^{atm\text{-}8}$/+), and its sibling (Sbl.) control (ctrl) at Day (D)7 and D35. *yw* data is the same data shown in Fig. 3B. *, *P*<0.05; **, *P*<0.01.
